# Notch2 inhibits proliferation of chronic myeloid leukemia cells

**DOI:** 10.3892/ol.2013.1159

**Published:** 2013-01-29

**Authors:** ZESONG YANG, CHUNXIU YANG, SHUNJUN ZHANG, YING LI, JIANBIN CHEN

**Affiliations:** 1 Department of Hematology, The First Affiliated Hospital of Chongqing Medical University, Chongqing;; 2 Department of Hematology, Affiliated Hospital of Zunyi Medical College, Zunyi;; 3 Department of Hematology and Rheumatism, Chongqing Three Gorges Central Hospital, Chongqing, P.R. China

**Keywords:** chronic myeloid leukemia, Notch signal, gene transfection, cell proliferation

## Abstract

The Notch signaling pathway has been shown to be involved in the progression of chronic myeloid leukemia (CML). The aim of this study was to investigate the effects of exogenous Notch2 overexpression on cell proliferation and possible mechanisms in the human CML cell line K562. When exogenous intracellular fragment of Notch2 (ICN2) was transfected into K562 cells with Lipofectamine™ 2000, the expression of Notch2 mRNA and protein were upregulated. Cell numbers decreased and the proliferation was inhibited significantly after transfection with ICN2. G1 phase cells increased and S phase cells decreased 48 h after transfection. Finally, the expression of Numb, Bcl-2, NF-κB and TGF-β1 was detected. It was found that the expression of NF-κB and TGF-β1 mRNA was increased, while Bcl-2 was downregulated, with Numb expression unchanged. Our study indicates that the Notch pathway is activated in K562 cells after ICN2 transfection. It inhibited the proliferation of K562 cells, likely by upregulating the expression of NF-κB and TGF-β1 mRNA and downregulating the expression of Bcl-2.

## Introduction

The Notch signaling pathway is a highly conserved evolutionarily signaling pathway which plays an important role in regulating the process of development in species as diverse as *Drosophila* to humans (1,2). The Notch signaling pathway is composed of a Notch receptor, ligand and CBF1/Su(H)/Lag-1 family (CSL) DNA binding protein. In mammals, four homolog Notch receptors (Notch 1–4) and five Notch ligands (Jagged 1 and 2, and Delta-like 1, 3 and 4) have been identified. Both Notch receptors and ligands are evolutionarily conserved single-pass transmembrane proteins. After Notch ligands bind to the receptors, the receptor undergoes at least two proteolytic cleavage events, releasing the Notch intracellular domain (NICD), which is activated into the cytoplasm, and then translocated to the nucleus, where it binds to the CSL (CBF1 in humans, RBP_J_ in mice, Suppressor of Hairless in *Drosophila* and Lag1 in *C. elegans*) protein (3). After binding to NICD, CSL turns from a transcription repressor to a transcriptional activator (3). The NICD/CSL complex then recruits the co-activator mastermind-like (MAML) and p300 proteins to form a ternary complex that subsequently activates the transcription of the Notch target genes (4,5). Canonical target genes of Notch include HES1, HES5 and Hey. The activated Notch signaling pathway determines the cell fate, maintains the stem cell state and affects cell proliferation, differentiation, apoptosis, organ formation and morphogenesis (6). In recent years, studies have found that abnormal Notch signaling is involved in tumor formation, hereditary diseases, autoimmune diseases and several other processes (7).

Several studies have demonstrated that Notch signaling is involved in the development of lymphoid leukemias (8), but only a few studies have revealed abnormal Notch expression in myeoloid leukemias. Whether abnormal Notch signaling can result in myelocytic leukemia remains unclear. Certain studies, however, have shown that Notch can affect myelopoiesis, with Notch ligands suppressing the differentiation of progenitor cells to myeloid cells (9). Notch2 overexpression is implicated in the development of chronic B-cell lymphoid leukemias, since B-cells are able to survive significantly longer than normal cells. A study by Ishiko *et al* found that Notch signaling did not affect the proliferation of the chronic myeloid leukemia cell line K562, but inhibited the development of erythroid/megakaryocytic cells by suppresing GATA-1 activity and inducing apoptosis in cooperation with 12-*O*-tetradecanoylphorbol-13-acetate (TPA) (10). In contrast, another study demonstrated that activated Notch signaling inhibited the growth of K562 cells, possibly by upregulating expression of Rb protein (11), although the precise mechanism is not clear.

In this study, K562 cells were used to observe the effects of overexpression of the intracellular domain of Notch2 (ICN2). Overexpression of ICN2 inhibited the proliferation of K562 cells and caused G1 arrest. Notably, the expression level of NF-κB and TGF-β1 genes were upregulated in K562 cells transfected with ICN2, and Bcl-2 was downregulated, but Numb expression was unchanged. These results may suggest that Notch signaling inhibits the proliferation of K562 cells, possibly by regulating gene expression.

## Materials and methods

### Cell culture and plasmid transfection

The human CML cell line K562 was propagated in RPMI-1640 medium (Hyclone, Logan, UT, USA) containing 10% fetal bovine serum (FBS) (Hyclone). The cell lines were grown in a humidified incubator at 37°C in the presence of 5% carbon dioxide. K562 cells were propagated every two days. One day before transfection, the cell medium was changed to ensure the cells were viable. Log phase growth cells were washed, resuspended in RPMI-1640, plated in 12-well plates at a density of 5×10^5^/ml and cultured for 1 h. Plasmid pcDNA3.1-ICN2 was transfected into the cells with Lipofectamine™ 2000 according to the manufacturer’s instructions (K562-ICN2). Vector pcDNA3.1 was used as control (K562-blank).

### Cell proliferation assay

Cells were seeded in 96-well plates at a density of 1,500 cells per well and transfected with ICN. Cell proliferation was assayed after 24 and 48 h of culture, by incubating in 20 *μ*l methylthiazole tetrazolium (MTT) solution. Cells were then incubated at 37°C for a further 6 h when 150 *μ*l of dimethyl sulfoxide (DMSO; Sigma, St. Louis, MO, USA) was added to each well and subsequently mixed at room temperature for 10 min. The spectrophotometric absorbance of the supernatant was measured at 550 nm. Each assay was repeated at least three times and data were analyzed with the LSD t-test.

### Cell cycle analysis

K562 cells were plated at a density of ∼3×10^5^/l in 12-well plates and transfected with plasmid for 48 h. Cells were collected, fixed in 1% methanol-free formaldehyde for 20 min and subsequently suspended in a 70% ethanol solution. Cells were then suspended in 1 ml of 0.1% Triton X-100 solution and incubated in 500 *μ*l propidium iodide solution (50 *μ*g/ml) containing 250 *μ*g of DNase-free RNase A. Cells were analyzed using fluorescence-assisted cell sorting (FACS). Each assay was repeated in triplicate and data were analyzed with the LSD-t test.

### RNA extraction and semiquantitative reverse transcription (RT)-polymerase chain reaction (PCR)

Total RNA was extracted from K562 cells with TRIzol reagent (Roche Diagnostics, Mannheim, Germany) according to the manufacturer’s instructions. cDNA was synthesized using a kit from Takara with oligo-dT as a primer. PCR was run for 35 cycles with cDNA from 0.1 *μ*g of total RNA as a template. The PCR products were resolved on 1.5% agarose gels and visualized by GoldView staining. The RT-PCR primers used in this study were: human Notch2 (5′ sense, TGGTGACCGAGATCCTG AAG; 3′ antisense, TTGTTCACAGAGCCTTGTTG); Hes1 (5′ sense, TAGCTGATCAGTGGCGTGAC; 3′ antisense, ATCATCTGGCCTAGGAGACC); Hey1 (5′ sense, GAGAGG TCCTCCATTGGAAT; 3′ antisense, ATGCACAACAAT GGCAACAG), Numb 5′ sense, TACCACGTCCTCACCTG TGG; 3′ antisense, TGAAGACTGCAGAACCATTG); Bcl-2 (5′ sense, ACCTGACCACTAGCCTCCTG; 3′ antisense, GCAGAGCACAGGATTCACAG); TGF-β1 (5′ sense, GCCTTGATGGAGAGCTTCAC; 3′ antisense, CTTGTG GTGGATGTGGACTG), NF-κB (5′ sense, AGTCTGTCC AGGCTCGTCAT; 3′ antisense, GGACAGGAAGCTCCT GAATG); and human β-actin I (5′ sense, ACTTGCGCA GAACAAGAGAT; 3′ antisense, ACTGCCGCCTTCTCC TTAGA); human β-actin II (5′ sense, GATCTGGCACCA CACCTTCT; 3′ antisense, AAGGAAGGCTGGAAGAGAGC).

### Western blot analysis

To detect the expression of Notch2, whole cell lysates were prepared using cell lysis buffer for western blot analysis and immunoprecipitation [20 mM Tris (pH 7.5), 150 mM NaCl, 1% Triton X-100, sodium pyrophoshate, β-glycerophosphate, EDTA, Na_3_VO_4_, leupeptin, 0.1 mM PMSF]. Cell extracts were collected by centrifugation at 13,000 × g for 8 min at 4°C. Protein concentrations of the cell extracts were determined by BCA protein assay reagents (Beyotime Institute of Biotechnology, Jiangsu, China), according to the manufacturer’s instructions. Proteins were resolved by SDS-PAGE on 10% polyacrylmide gel. The proteins were transferred to a PVDF membrane and detected using immunoblotting. The Notch2 antibody was incubated with the membrane for 12 h at 4°C, and immunoreactive proteins were visualized by incubation with a goat anti-mouse immunoglobulin conjugated to horseradish peroxidase (HRP), and the membrane was developed using Pro-light HRP chemiluminescence reagents from Tiangen Biotech (Beijing) Co., Ltd., Beijing, China. All antibodies were purchased from Santa Cruz Biotechnology Inc. (Santa Cruz, CA, USA).

### Statistical analysis

To analyze the data, the mean ± SD was calculated. One-way ANOVA was used to compare the mean values among multiple groups, and the t-test was applied to compare the mean values between pairs of groups. All data was processed by SSPS 10.0 (SPSS, Inc., Chicago, IL, USA). P<0.05 was considered to indicate a statistically significant result.

## Results

### Notch2 gene is overexpressed successfully in K562 cells

**Figure 1:**
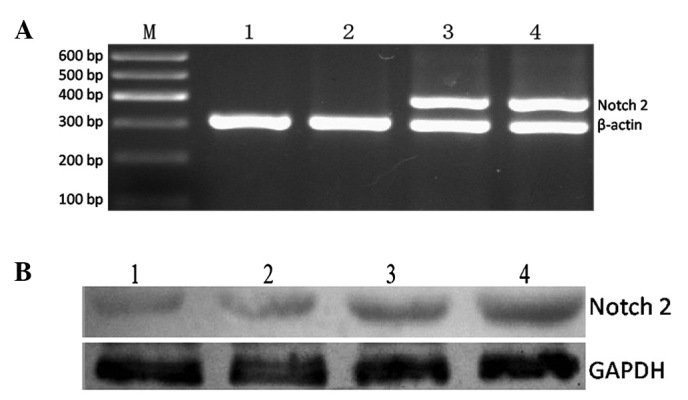
Expression of Notch2 mRNA in K562 cells. M, DNA Marker I; lanes 1 and 2, K562-blank at 24 and 48 h; lanes 3 and 4, K562-ICN2 at 24 and 48 h.

To confirm that the plasmid pcDNA3.1-ICN2 was successfully introduced to the K562 cells, RT-PCR and western blot analysis were employed to detect the expression of the gene and protein, respectively. As shown in Fig. 1, the expression of Notch2 mRNA and protein was upregulated significantly, suggesting that ICN2 was successfully introduced into K562-ICN2 cells.

### Notch2 signaling is activated in K562 cells

**Figure 2:**
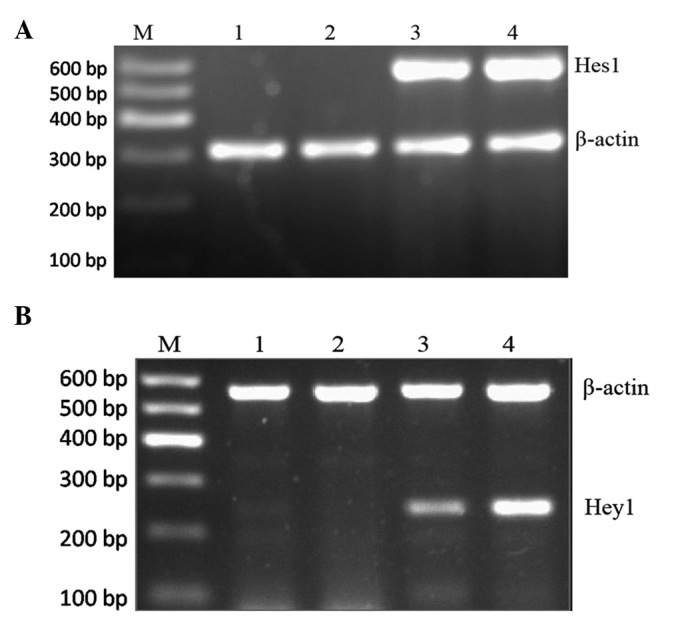
(A) Expression of Hes1 mRNA in K562 cells after transfection with ICN2. M, DNA Marker I; lanes 1 and 2, K562-blank at 24 and 48 h; lanes 3 and 4, K562-ICN2 at 24 and 48 h. (B) Expression of Hey1 mRNA in K562 cells after transfection with ICN2.

To further assess the activation of the Notch signaling pathway, RT-PCR was used to detect the expression of Notch target gene Hes1, and Hey1 of the transfected and control group. As shown in Fig. 2, Hes1 and Hey1 were not expressed in K562-blank cells, but they were expressed strongly in the K562-ICN2 group, indicating that Notch signaling was activated in K562-ICN2 cells.

### Overexpression of ICN2 changes the morphology of K562 cells

**Figure 3:**
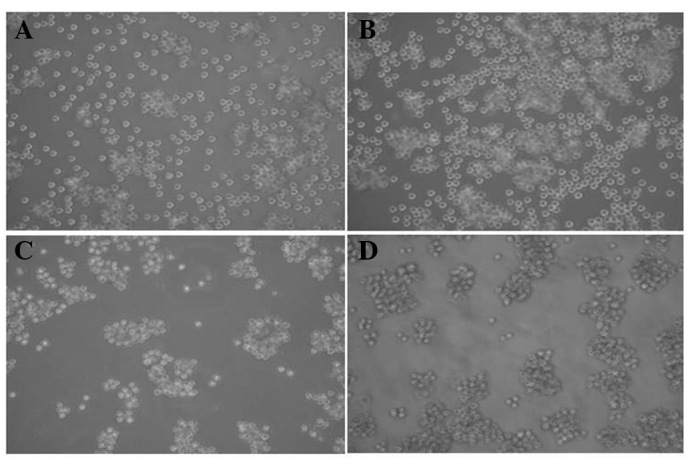
Morphology of K562 cells after transfection with ICN2 under inverted phase contrast microscope (ICC, ×400. (A) and (B) K562-blank group at 24 and 48 h; (C) and (D) K562-ICN2 group at 24 and 48 h.

As observed under an inverted phase contrast microscope (Fig. 3), the plasmalemma of K562-blank cells was transparent. The longer the culture time, the larger the cell number. In contrast, the K562-ICN2 cells were aggregated when cultured for 24 h with small cells with a cloudy plasmalemma. The longer the culture time the smaller the cell diameter and volume.

### Activation of Notch by transfection with ICN2 inhibits the proliferation of K562 cells

**Figure 4:**
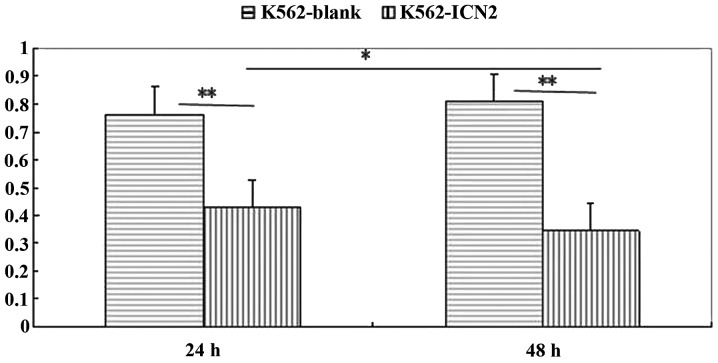
Cell proliferation of K562 cells after transfection with ICN2. ^*^P<0.05, ^**^P<0.01.

To determine whether Notch signaling affects the proliferation of K562 cells, the growth of K562-ICN2 cells was compared with the K562-blank cells using the MTT assay. As shown in Fig. 4, the growth of K562-ICN2 cells was considerably slower than that of the K562-blank cells (P<0.01). These data suggest that overexpression of the constitutively active Notch signaling could inhibit the proliferation of the human CML cell line K562 *in vitro*.

### Notch activation rearranges the distribution of the cell cycle of K562 cells

**Figure 5:**
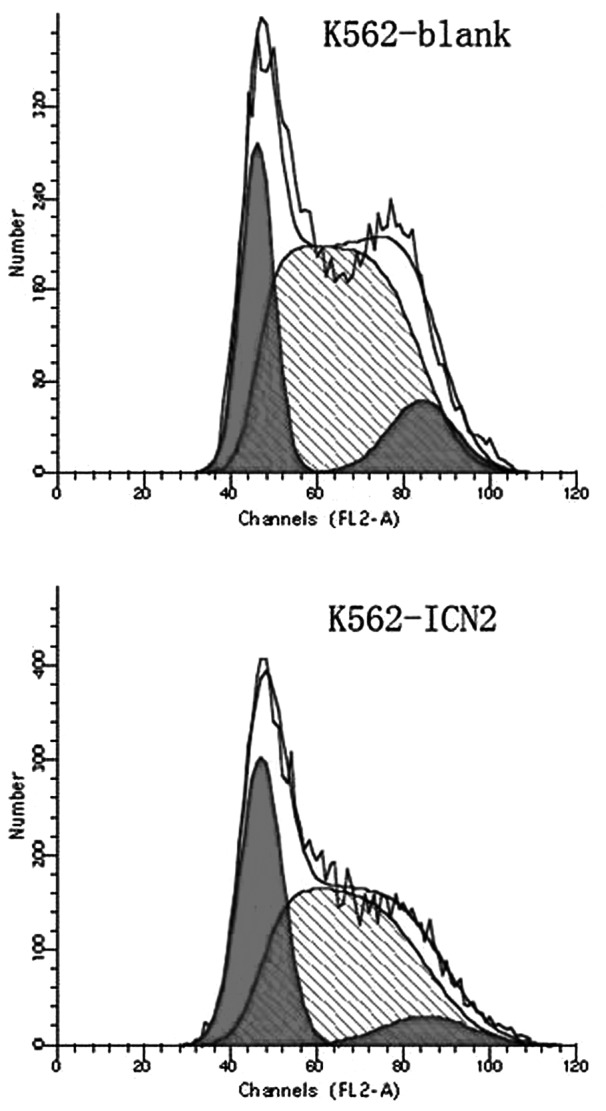
Cell cycle results of K562 cells.

The effect of transfection with ICN2 for 48 h on the course of the cell cycle using flow cytometry was investigated. As shown in Fig. 5 and Table I, K562 cells showed a G1 arrest and there were fewer S phase cells.

### Activation of Notch signaling pathway regulates the expression of certain genes in K562 cells

The molecular mechanism underlying the growth-regulatory effect of Notch signaling on K562 was investigated. The expression of the molecules related with cell proliferation in K562 cells was measured. RT-PCR analysis showed that the expression of NF-κB and TGF-β1 was upregulated, and that the expression of Bcl-2 was downregulated. The expression of Numb, however, was unchanged.

## Discussion

Different Notch receptors have varying functions in the development of tumors, depending on the cell context. Notch1 has been shown to inhibit the cell growth of hepatic carcinoma, small cell lung cancer and prostate cancer in a previous study (7). When all four Notch receptors are activated they inhibit the growth of acute B-cell lymphoid leukemia and induce apoptosis (12). In contrast, one study has proven that overexpression of Notch3 promots the growth of human lung cancer cells *in vitro* and inhibits the differentiation of lung cancer cells in transgenic mice. Notch3 is expressed at a high level in human pancreatic cancer cells and acute T-cell lymphoid leukemia cells. The mechanism of the Notch receptor function as an oncogene or tumor suppressor gene remains to be elucidated.

An important member of the Notch signaling family is the Notch2 receptor, which plays a key role in regulating developmental processes in the embryo (13). A study has shown that Notch2 was upregulated in non-small cell lung carcinoma (NSCLC) and promoted the proliferation of NSCLC cells (14). Expression of Notch2 is necessary for marginal zone B-cell development and is related to CD23 overexpression in chronic B-cell lymphoid leukemia (15). Overexpression of ICN2 can induce T-cell lymphoid leukemia and cells are more easily prone to becoming CD8 cells when expression of Notch2 is inhibited. Conversely, Notch2 is a new tumor suppressor; it inhibits tumor progress in human breast cancer and small cell lung cancer (16). In this study, activation of the Notch2 signal pathway significantly inhibited the proliferation of K562 cells and changed the cell morphology. These results suggest that Notch2 gene is a potential tumor suppressor in chronic myeloid leukemia.

Tumor cells cleave fast and have a high proliferation ability. G1 phase is a DNA presynthetic phase of cells which determines the cell cycle. A recent study found the p53 gene product is an important factor in the regulation of the cell cycle and apoptosis, and p53 accumulated quickly when DNA was damaged by extrinsic factors and G1 arrest occurred (17). Notch2 inhibited the proliferation of K562 cells and may simulate the function of p53 to induce G1 arrest.

The crosstalk between Notch and other signaling pathways is complicated, and the physiological correlation remains unclear. Numb was considered to be a security device of WNT-Notch signal transduction pathway. The silence, loss of function or mutation of this gene can induce tumorigenesis (18). A study has found that there is crosstalk between Notch and NF-κB when their roles in normal development and cancer formation are considered (19). Transfected Notch1-ICD in the mouse T-ALL cell line stimulates NF-κB expression through a canonical pathway. A study on a human cervical cancer cell line found that Notch1 activates NF-κB by interacting with the IκB kinase (IKK) signal. This occurred in both the cytoplasm and the nucleus (20). The Bcl-2 family of proteins determined the mitochondrial events in cell death and mediated the apoptosis induced by a numner of stimulants (21). Bcl-2 is expressed at a high concentration in many tumor cells and is involved in drug resistance. TGF-β1 is a pleiotropic anti-inflammatory factor, which regulates T-cell differentiation. When γ-secretase inhibitor (GSI) inhibits Notch signaling, TGF-β1 induces Foxp3 expression and naive T-cell proliferation is blocked (22). TGF-β1 inhibits the growth of normal cervical cells causing G1 arrest and apoptosis (23).

In this study, it was identified that overexpression of ICN2 upregulated the expression of NF-κB and TGF-β1, and downregulated the expression of Bcl-2, while the expression of Numb was unaffected. These results suggest that enhanced expression of Notch2 inhibits cell proliferation, suggesting a pathway which may affect the cell cycle distribution by upregulating the NF-κB and TGF-β1 genes and downregulating Bcl-2. Further research is required to confirm our findings and to elucidate the mechanism of Notch in chronic myeloid leukemia.

## Figures and Tables

**Figure 6:**
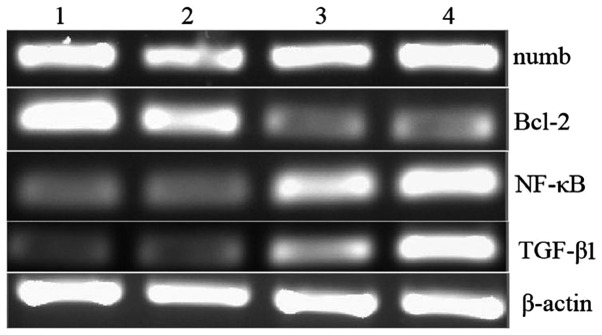
Expression of related genes in K562 cells after transfection with ICN2. Lanes 1 and 2, K562-blank at 24 and 48 h; lanes 3 and 4, K562-ICN2 at 24 and 48 h.

**Table I:** Cell cycle change of K562 cells after transfection with ICN2 for 48 h (mean ± SD, %).

Group	G1 phase	S phase	G2+M phase	CV value
K562-blank	21.52±2.51	63.37±3.44	10.82±0.96	4.96
K562-ICN2	39.17±1.50^a^	32.94±3.75^a^	13.41±1.97	4.73

a P<0.01; compared with the control group.
